# Exploring road safety using alignment perspective features in real driving images: A case study on mountain freeways

**DOI:** 10.1371/journal.pone.0305241

**Published:** 2024-06-17

**Authors:** Shijian He, Hongmei Fu, Jie Wang, Jiacheng Yang, Yanqing Yao, Jiaojiao Kuang, Xiangliang Xiao

**Affiliations:** 1 School of Traffic and Transportation Engineering, Changsha University of Science and Technology, Changsha, China; 2 International College of Engineering, Changsha University of Science and Technology, Changsha, China; 3 Hunan Communications Research Institute Co., Ltd, Changsha, Hunan, China; Nanjing Forestry University, CHINA

## Abstract

**Introduction:**

While driving, drivers frequently adapt their driving behaviors according to their perception of the road’s alignment features. However, traditional two-dimensional alignment methods lack the ability to capture these features from the driver’s perspective.

**Method:**

This study introduces a novel method for road alignment recognition, employing image recognition technology to extract alignment perspective features, namely alignment perspective skewness (APS) and alignment perspective kurtosis (APK), from in-real driving images. Subsequently, the K-means clustering algorithm is utilized for road segment classification based on APS and APK indicators. Various sliding step length for clustering are employed, with step length ranging from 100m to 400m. Furthermore, the accident rates for different segment clusters are analyzed to explore the relationship between alignment perspective features and traffic safety. A 150 km mountain road section of the Erlianhaote-Guangzhou freewway from Huaiji to Sihui is selected as a case study.

**Results:**

The results demonstrate that using alignment perspective features as classification criteria produces favorable clustering outcomes, with superior clustering performance achieved using shorter segment lengths and fewer cluster centers. The road segment classification based on alignment perspective features reveals notable differences in accident rates across categories; while traditional two-dimensional parameters-based classification methods fail to capture these differences. The most significant differences in accident rates across categories are observed with segment length of 100m, with the significance gradually diminishing as segment length increases and disappearing entirely when the length exceeds 300m.

**Implication:**

These findings validate the reliability of using alignment perspective features (APS and APK) for road alignment classification and road safety analysis, providing valuable insights for road safety management.

## 1 Introduction

Traffic accidents are a global safety issue that plagues contemporary human society. They are not only one of the primary causes of human fatalities but also account for economic losses amounting to 10%-12% of the global GDP [1]. Although accidents may exhibit some degree of randomness, researchers acknowledge that inherent flaws in road alignment can elevate the risk of accidents. Among the numerous inherent features of roads, geometric alignment emerges as one of the crucial factors influencing road safety. Its direct influence on guiding drivers’ driving direction and its strong correlation with vehicle stability have made it a central focus of research [2].

Road segments are primarily classified based on two-dimensional geometric (horizontal and vertical) parameters, and road safety analysis is conducted based on these geometric features. A large body of existing research also has confirmed that the horizontal curves radius [3–6], vertical gradients [7–10], and their combined changes [11, 12] significantly affect road traffic safety. The operating speed theory, introduced by Hassan [13], is the predominant framework utilized for alignment safety assessment. It includes models that outline operating speeds across different alignment categories and clarifies the relationship between operating speed and accident rates. However, recent studies have indicated that categorizing the road alignment solely based on two-dimensional horizontal and vertical features overlooks the micro-variations in spatial geometric characteristics of the alignment [14]. The road alignment is essentially a three-dimensional spatial curve. Road designers always chose to represent the three-dimensional spatial parameters using two-dimensional horizontal and vertical parameters, mainly because of the complexity involved in solving three-dimensional parameters. Recently, some scholars [15] have attempted to use three-dimensional parameters, such as spatial curvature and torsion, to assess road alignment safety. They have discovered that the locations of continuous changes in three-dimensional parameters are directly associated with accident-prone sections. On this basis, Ahmed [16] and Wang [17] used three-dimensional parameters to establish a Bayesian accident prediction model, and found their model outperformed traditional two-dimensional parameters-based models. These findings demonstrate that road segment classification and prediction models, based on three-dimensional parameters closely reflecting their intrinsic features, yield more effective and reliable modeling results.

However, it’s crucial to acknowledge that regardless of whether two-dimensional or three-dimensional parameters are utilized for road segment classification, there’s a departure from the fundamental design principle of "products should meet user needs". When it comes to road alignment, which provides drivers with the fundamental direction of driving, its classification and evaluation should be based on the driver’s perspective. Wang et al. [18] studied the subjective classification of road geometric features by middle-aged drivers. This study found that drivers use visual features of road alignment to identify subjective categories, rather than the specific horizontal and vertical geometric parameters typically considered by designers. While driving, drivers visually observe the scene of the road ahead, which is projected onto their retinas, forming the alignment perspective views. In fact, the perspective view is considered a critical method for evaluating the effectiveness of road alignment design, since it can reflect the intrinsic characteristics of road alignment from the driver’s perspective [19, 20].

Currently, the geometric features of alignment perspective views lack a unified quantitative description, with methods reflecting the intrinsic characteristics of alignment in drivers’ visual perception predominantly confined to qualitative analysis [21]. Therefore, there is a need to explore a quantitative computational tool to uncover the underlying geometric parameters within perspective views. Recently, in the field of image recognition, quantitative scientific computational tools for morphological feature analysis based on mathematical morphology theory are rapidly advancing [22]. Several quantification metrics have been proposed to describe the intrinsic shape or alignment characteristics with similar invariance, ensuring consistent depiction of shape or algnment features of the same object from different perspectives. These metrics have found wide applications in various domains such as image recognition [23, 24] and road landscape feature analysis [25], yielding favorable outcomes. These studies have provided insights into methods for extracting alignment parameters from perspective views. This form of similarity invariance conveniently enables a consistent depiction of linear perspective view features of the same cross-section from different viewpoints, potentially aiding in addressing the challenge of unified quantitative expression of alignment features in perspective views.

Therefore, this study aims to explore and achieve two main objectives: 1) utilizing driving scene images, which offer a real-time and convenient perspective synchronized with driving tasks, we employ morphological quantitative computational tools to uncover invariant parameters of road surface contours from these images.This methodology introduces alignment perspective feature parameters (APS and APK) based on the driver’s viewpoint. 2) examining whether these novel alignment feature parameters, rooted in the driver’s perspective, prove more efficacious in road segment classification and safety analysis compared to traditional methods. To achieve these objectives, this study first employs on-road driving experiments to capture consecutive, clear vehicle images as drivers traverse different lanes from the same perspective. Then, it uses image recognition algorithms such as edge detection to obtain alignment perspective feature parameters with similarity invariance from the driving images. Based on obtained alignment perspective parameters, the K-means clustering algorithm is applied to classify road segments. Last, by comparing the efficacy of the new classification results with traditional methods in analyzing road segment safety, we demonstrate the reliability and engineering practicality of this new approach.

## 2 Method

### 2.1 Data collection

This study collects driving video data from a 150km stretch of the G55 Er-Guang Freeway in Guangdong Province, from Huaiji to Sihui in both two directions, and obtains accident data from January 2016 to December 2018 for our analysis. The experimental road section passes through 15 tunnels, 3 bridges, and 6 interchanges, and the selected section of this highway is paved with asphalt, featuring a design standard of six lanes with three lanes in each direction, a design speed of 100 km/h, lane widths of 3.75m, a maximum longitudinal slope of 4.5%, a minimum radius of 660m for horizontal curves, and a minimum radius of 8000m for vertical curves. The annual average daily traffic was 26,526 standard vehicles during the accident statistics period (Table 1).

**Table 1:** Design information of experimental road segment.

Design Parameters	Value
The length of freeway (km)	150
Design Speed (km/h)	100
Maximum horizontal curve radius (m)	5400
Minimum horizontal curve radius (m)	660
Maximum radius of vertical curve (m)	80000
Minimum radius of vertical curve (m)	8000
Maximum vertical gradient (%)	4.50
Minimum vertical gradient (%)	0.40
The number of straight and gentle curve segment	482
The number of gentle horizontal curve segment	91
The number of combined horizontal and vertical curve segment	18
The number of gradient segment	93

**Fig 1:**
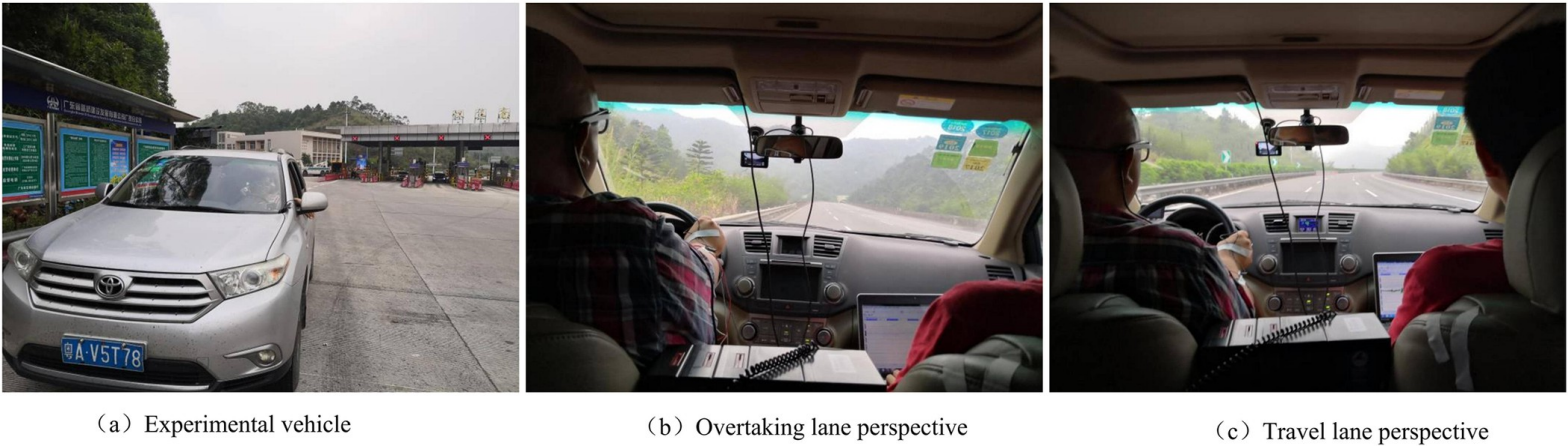
Experimental vehicle and driving video collection.

The driving image extraction experiment is conducted in daylight and under clear weather and good visibility conditions. The daytime road scene is captured from a fixed angle using a dashboard camera mounted on the experimental vehicle (a 2016 Toyota Highlander). The camera has a frame rate of 24 frames per second and a resolution of 960×720 pixels. It is positioned at a viewing height of 1.5m to record the driving image of the road ahead. The dimension of the vehicle is 4.890m in length, 1.925m in width, and 1.725m in height, as depicted in Fig 1A. This study collects continuous driving video from two different perspectives: the overtaking lane and the travel lane (Fig 1B and 1C).

### 2.2 Extraction of alignment perspective features

**Fig 2:**
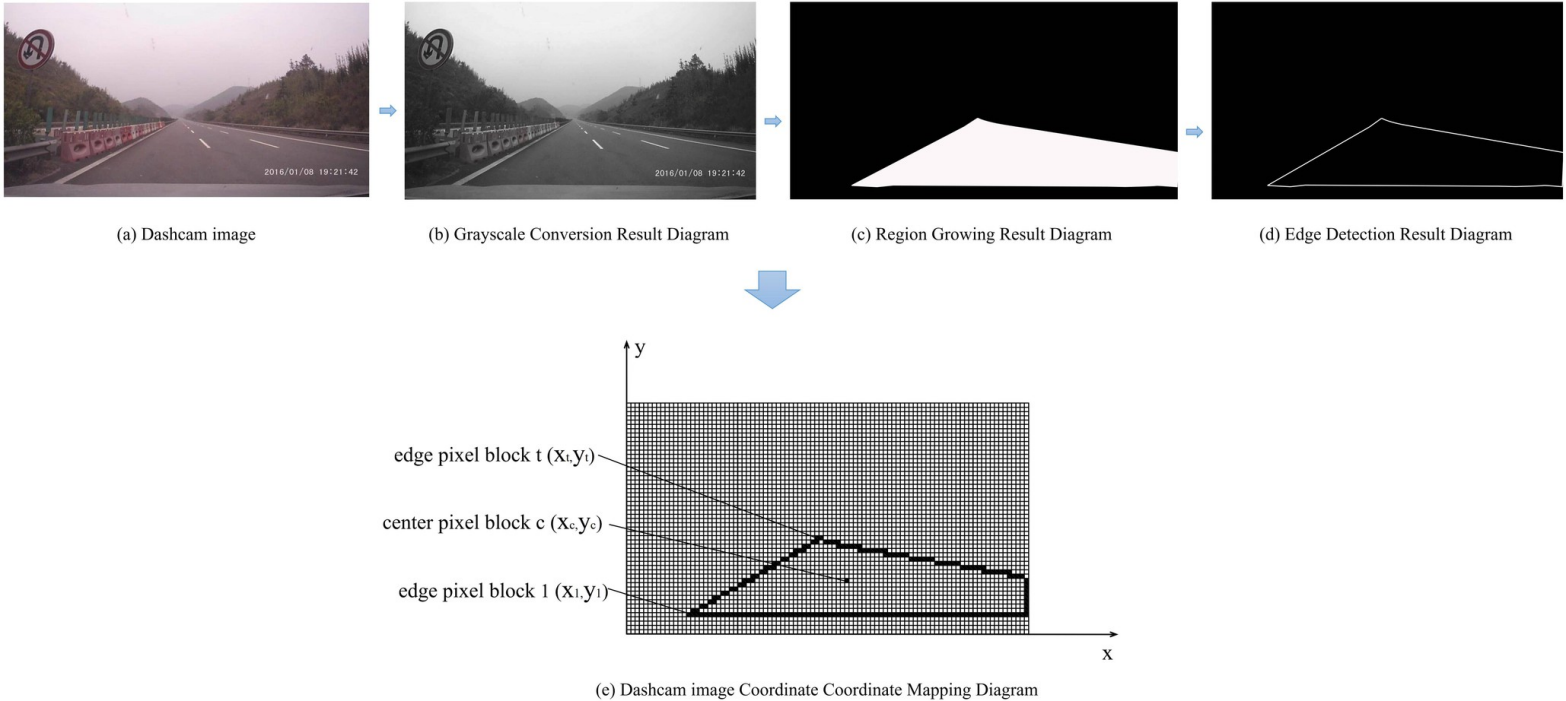
The process of extracting alignment perspective features.

This study utilizes a fixed-length method to process perspective image from driving video. It extracts one frame image for the driving video for every 5 m, thereby obtaining consecutive and clear driving images depicting road cross-section ahead, including the shape of road surface. The image processing procedure is shown in Fig 2. Specifically, first, the driving image is converted to grayscale (Fig 2B). Second, the region-growing algorithm is used to identify the visible road surface area (excluding emergency lanes) in the driving images, resulting in binarized driving images displaying only black and white colors (Fig 2C). Third, the Canny algorithm is applied for edge detection to obtain driving images showing only the edges of the visible road surface area (Fig 2D). Forth, a Cartesian coordinate system is introduced into the edge detection result image, where each pixel block is assigned corresponding coordinates. With the pixel block in the bottom-left corner as the origin, a Cartesian coordinate system is established with the horizontal direction (parallel to the horizontal road cross-section) as the x-axis and the vertical direction (perpendicular to the horizontal road cross-section) as the y-axis. A schematic diagram of the Cartesian coordinate system established based on the driving images is shown in Fig 2E.

In Fig 2E, (*x_t_, y_t_*) is the coordinates of the t-th edge pixel block that forms the edge line of the road alignment contour. It is numbered in a clockwise direction starting from the first pixel block on the left. (*x_c_, y_c_*) is the coordinates of the c-th center pixel block, which is located at the same horizontal position as the edge pixel blocks and at the center of the alignment contour. The central distance between edge pixel blocks and center pixel blocks is calculated based on the coordinates of the pixel blocks, that is:

$$P(t)=\sqrt{{\left(x_{t}-x_{c}\right)}^{2}-{\left(y_{t}-y_{c}\right)}^{2}}$$ (1)

where *P*(*t*) is the central distance between the t-th edge pixel block and the c-th center pixel block.

Considering the central distances of pixel blocks, the r-th order raw moments and the r-th order central moments of the set of central distances for each pixel block are calculated. The formulas for raw moments and central moments are as follows:

$$m_{r}=\frac{1}{n}\sum_{t=1}^{n}{\left[P(t)\right]}^{r}$$ (2)

$$C_{r}=\frac{1}{n}\sum_{t=1}^{n}{\left[P(t)-m_{1}\right]}^{r}$$ (3)

where n is the total number of edge pixel blocks, m_r_ is the r-th order raw moment of the central distance set for all pixel blocks, C_r_ is the r-th order central moment of the central distance set for all pixel blocks.

Since the numerical values of the r-th order raw moments and r-th order central moments of the central distance set of pixel blocks can represent the information of shape changes in the perspective view of alignment, this study calculates two similarity invariants: skewness [26] and kurtosis [27]. Skewness measures the asymmetry of the distribution of the central distance set between edge pixel blocks and center pixel blocks, incorporating the mathematical definition of skewness, this study calculates the Alignment Perspective Skewness (APS), which is a measure of the tilt or symmetry of the road alignment shape from the driver’s perspective, as calculated by Eq 4. Kurtosis measures the sharpness of the distribution of the central distance set between edge pixel blocks and center pixel blocks, incorporating the mathematical definition of kurtosis, this study calculates the Alignment Perspective Kurtosis (APK), which is a measure of the sharpness or flatness at the top of the road alignment shape from the driver’s perspective, as calculated by Eq 5.


$$APS=\frac{C_{3}}{C_{2}^{3/2}}=\frac{\frac{1}{n}\sum_{t=1}^{n}{\left[P(t)-\frac{1}{n}\sum_{t=1}^{n}P(t)\right]}^{3}}{{\left[\frac{1}{n}\sum_{t=1}^{n}{\left[P(t)-\frac{1}{n}\sum_{t=1}^{n}P(t)\right]}^{2}\right]}^{3/2}}$$ (4)

$$APK=\frac{C_{4}}{C_{2}^{2}}=\frac{\frac{1}{n}\sum_{t=1}^{n}{\left[P(t)-\frac{1}{n}\sum_{t=1}^{n}P(t)\right]}^{4}}{{\left[\frac{1}{n}\sum_{t=1}^{n}{\left[P(t)-\frac{1}{n}\sum_{t=1}^{n}P(t)\right]}^{2}\right]}^{2}}$$ (5)

## 3 Results and discussion

### 3.1 Verification of the similarity invariance of alignment perspective features

**Fig 3:**
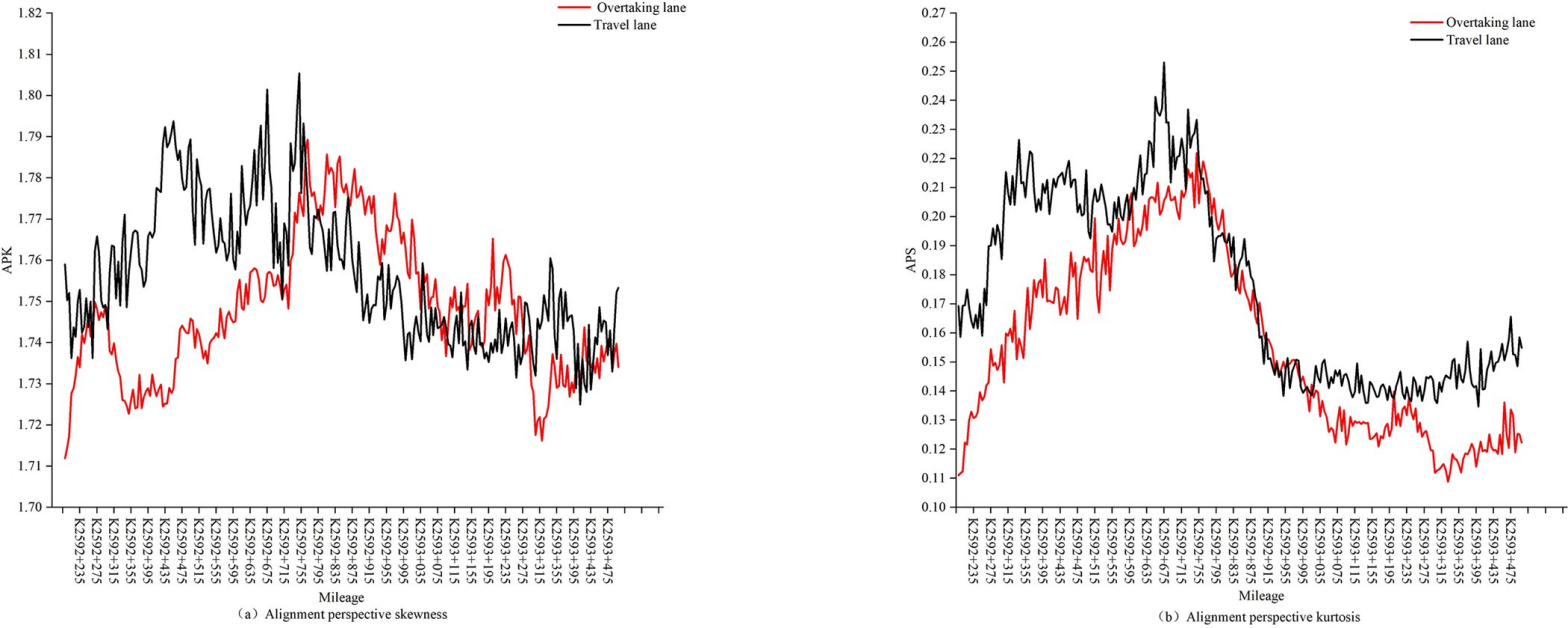
Statistical chart of alignment perspective feature indicators.

Across different lanes within the same cross section, drivers may observe varying alignment perspective features due to differences in viewing angles. This study utilizes the invariance of skewness and kurtosis to achieve unified description of alignment perspective features from different lane positions, thus requiring validation of the extracted indicators through similarity invariance test. To concretely illustrate the essence of the similarity invariance test, the variation patterns of APS and APK under the viewpoints of the overtaking lane and the travel lane in the sample segment from K2592+200 to K2593+400 are depicted in Fig 3. This study employs the Chebyshev distance [28] to quantitatively assess the similarity of these variation patterns.

As shown in Table 2, we consider the alignment perspective features of the overtaking lane and the travel lane as vectors and use the Chebyshev distance to calculate the distance between these two vectors as a basis for judging similarity invariance. When the Chebyshev distance between vectors is less than 0.1, it indicates that the difference between them is low, and they are considered similar [29]. The average Chebyshev distances between the vectors of the overtaking lane and the travel lane are for all road segment increments is less than 0.1, with the average similarity scores (ranging from [0,1]) is also very close to 1, indicating that the extracted perspective features in this study exhibit highly similar variation patterns across different lanes.

**Table 2:** Results of similarity invariance test.

Segment length(m)	The number of segment	APS				APK			
		Chebyshev distance		Similarity score		Chebyshev distance		Similarity score	
		Mean value	Standard deviation	Mean value	Standard deviation	Mean value	Standard deviation	Mean value	Standard deviation
100	12	0.044	0.028	0.958	0.025	0.037	0.013	0.964	0.012
200	6	0.041	0.018	0.961	0.017	0.042	0.014	0.960	0.013
300	4	0.045	0.018	0.957	0.017	0.044	0.017	0.958	0.015
400	3	0.045	0.018	0.957	0.017	0.044	0.017	0.958	0.015

Note: The range of Chebyshev distance is [0, +∞), with 0 signifying that the two vectors are identical. The smaller the distance, the greater the similarity between the vectors.

### 3.2 Categorization of road segments based on alignment perspective features

Classifying road segments according to the real alignment features observed by drivers is crucial for improving road alignment design and road safety management. In this regard, we attempt to utilize the aforementioned alignment perspective features (APS and APK) for road segment classification and evaluate the disparities between these results and those obtained through traditional classification methods.

Based on APS and APK indicators, this study utilizes the sliding step length method to categorize the experimental road segment, with step lengths of 100m, 200m, 300m, and 400m. Within each road segment unit, alignment perspective features are extracted every 5m. The average alignment perspective skewness (referred to as AAPS) and the average alignment perspective kurtosis (referred to as AAPK) within each road segment unit are used as classification indicators. Then we employ the K-means clustering algorithm [30] to cluster the road segments of various step lengths.

To assess the clustering effects, three common internal clustering evaluation indexes are used: Silhouette Coefficient (SC), Davies-Bouldin (DB), and Calinski-Harabasz (CH). The value of SC ranges between -1 and 1, with values closer to 1 indicating that the intra-cluster distance is closer and the inter-cluster distance is farther, implying a better clustering effect. A smaller value of DB signifies closer intra-cluster distances and farther inter-cluster distances, indicating a better clustering effect. A higher value of CH indicates higher intra-cluster cohesion and better inter-cluster separation, implying a better clustering effect. In Table 3, a significant decrease in the CH value as the step length increases suggests a worsening of the clustering effect when the step length of the road segment increases. Furthermore, with an increase in the number of cluster centers, the values of both SC and CH decrease, indicating that as the number of clusters increases, the clustering effect gradually deteriorates.

**Table 3:** Clustering results based on AAPS and AAPK.

Step length(m)	Number of cluster centers	Number of segments	Cluster Order	Cluster Centroid Coordinates		SC	DB	CH
				AAPS	AAPK			
100	2	533	K1	0.17	1.77	0.46	0.84	1200.93
		966	K2	0.12	1.74			
	3	588	K1	0.11	1.73	0.39	0.80	1077.94
		163	K2	0.2	1.79			
		748	K3	0.14	1.76			
	4	753	K1	0.14	1.75	0.40	0.87	1106.59
		481	K2	0.11	1.72			
		8	K3	0.27	1.94			
		257	K4	0.18	1.78			
200	2	269	K1	0.16	1.77	0.46	0.84	698.73
		480	K2	0.12	1.74			
	3	354	K1	0.14	1.76	0.40	0.84	679.47
		299	K2	0.11	1.73			
		96	K3	0.19	1.78			
	4	197	K1	0.11	1.72	0.36	0.90	624.32
		197	K2	0.16	1.77			
		47	K3	0.21	1.79			
		308	K4	0.13	1.75			
300	2	330	K1	0.12	1.74	0.46	0.83	453.81
		169	K2	0.17	1.77			
	3	263	K1	0.14	1.75	0.40	0.83	447.40
		155	K2	0.11	1.72			
		81	K3	0.19	1.78			
	4	105	K1	0.1	1.72	0.38	0.87	434.98
		228	K2	0.13	1.75			
		137	K3	0.16	1.77			
		29	K4	0.21	1.79			
400	2	239	K1	0.12	1.74	0.46	0.84	345.37
		135	K2	0.17	1.77			
	3	142	K1	0.11	1.73	0.41	0.85	327.69
		180	K2	0.14	1.76			
		52	K3	0.19	1.78			
	4	90	K1	0.16	1.77	0.37	0.95	294.01
		155	K2	0.13	1.75			
		108	K3	0.11	1.72			
		21	K4	0.21	1.79			

**Fig 4:**
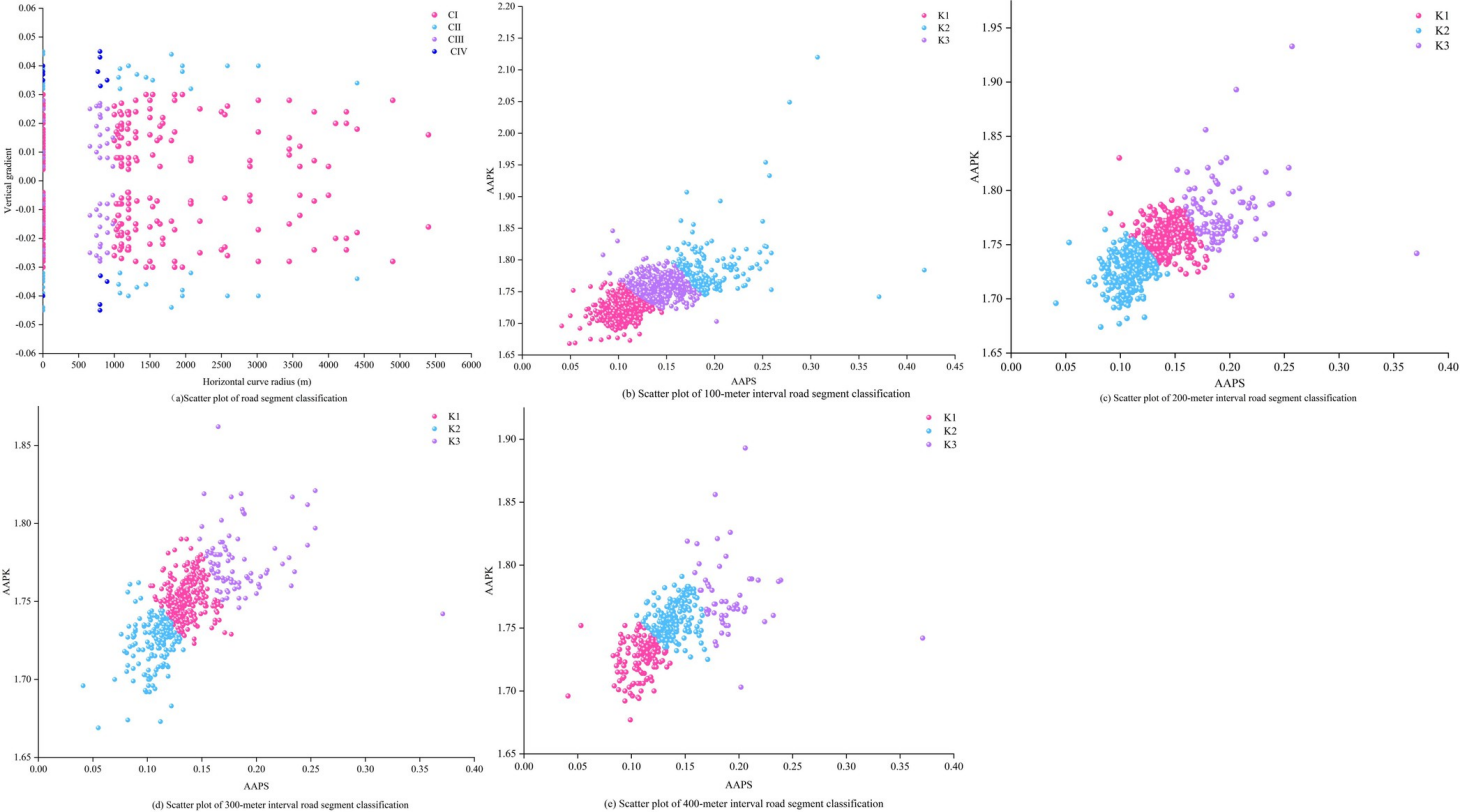
Scatter plot of clustering results for road segments at various step lengths.

We further examine the variations in segment categorization effects based on proposed perspective alignment features and traditional two-dimensional geometric parameters. Clustering results of three cluster centers under various step lengths from 100 to 400 meters are selected for comparison with traditional classification results, as shown in Fig 4. According to the traditional classification method and standards [31] based on two-dimensional geometric parameters of horizontal curvature and vertical gradient, the selected freeway can be divided into four categoryies of segment units: Category C-I (straight and gentle curve segments: horizontal curve radius > 1000m, vertical gradient < 3%), Category C-Ⅱ (gentle horizontal curve segments: horizontal curve radius ≤ 1000m, vertical gradient < 3%), Category C-Ⅲ (combined horizontal and vertical curve segments: horizontal curve radius > 1000m, vertical gradient ≥ 3%), and Category C-Ⅳ (gradient segments: horizontal curve radius ≤ 1000m, vertical gradient ≥ 3%). Fig 4A depicts the scatter plot of segment category distribution based on two-dimensional geometric parameters, where distances between segments of the same category are far, and different categories of road segments are intermixed and scattered without pattern. Fig 4B–4E display the distribution outcomes of segment categories with three cluster centers at different step lengths, classified by alignment perspective features (AAPS, AAPK). These figures illustrate clear categories with well-defined boundaries. Indeed, it is evident that the classification efficacy derived from alignment perspective features (AAPS, AAPK) surpasses that of the traditional classification method.

**Fig 5:**
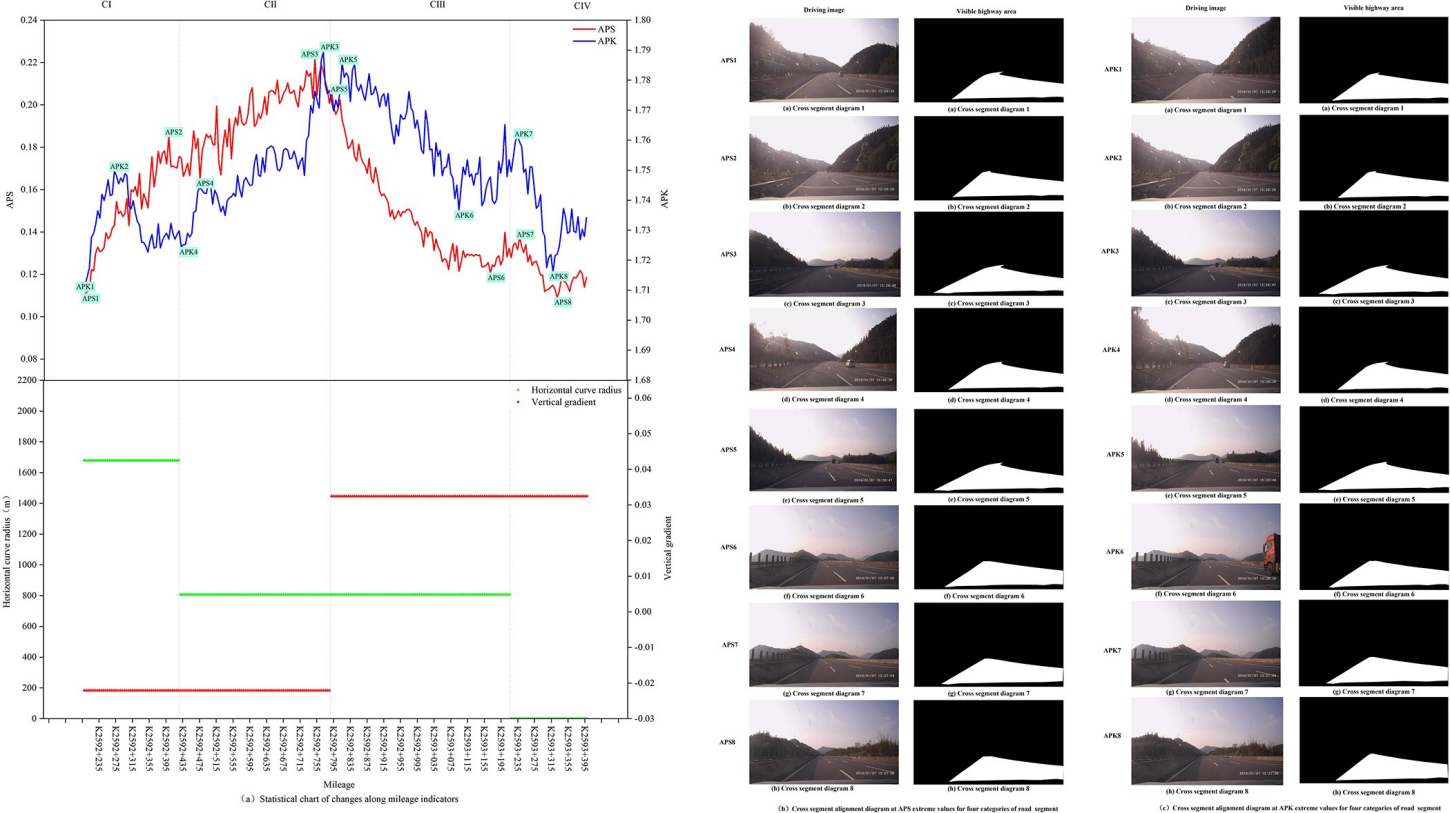
The comparison between alignment perspective features and traditional geometric parameters.

The road alignment observed by drivers is constantly changing during driving. Compared to traditional two-dimensional geometric parameters, APS and APK provide more real-time information about the changes in road shape that occur during driving. This is exemplified using the sample of the 1.2km selected freeway section of K2592+200 to K2593+400. Fig 5 illustrates the comparison between alignment perspective features and traditional geometric parameters. Specifically, in Fig 5A, we can observe that traditional methods segment C-I to C-IV with fixed horizontal and vertical alignment indicators for each segment, whereas APS and APK exhibit continuous changes. APK1, APK3, APK5, and APK7 respectively represent the maximum values of alignment perspective skewness (APK) for segment categories C-I to C-IV, while APK2, APK4, APK6, and APK8 represent the minimum values of APK for segment categories C-I to C-IV. Likewise, APS1 to APS8 represent the extremes of alignment perspective skewness (APS) for segment categories C-I to C-IV. Fig 5B and 5C depict driving images and road surface alignment extraction results for APS and APK extreme values in segments C-I to C-IV, respectively, with corresponding feature values listed in Table 4. For segments of the same classifications based on two-dimensional geometric parameters, there exists a considerable disparity between the maximum and minimum extremes of APS; while the disparity between the high and low extremes of APK is relatively low. As previously mentioned, the higher the APS value, the stronger the asymmetry of the road surface lineament. For instance, within the segment category of C-III, the maximum APS value (APS5 = 0.206) at segment diagram K2592+790 indicates a more asymmetric road surface alignment compared to the minimum APS value (APS6 = 0.121) at segment diagram K2593+170.

**Table 4:** Extreme values of alignment perspective features for different categories of road segments.

Segment category	Horizontal curve radius (m)	Vertical gradient	APS				APK			
			Number	Mileage	Extreme	Value	Number	Mileage	Extreme	Value
C-Ⅰ	1679	-0.022	APS1	K2592+	Maximum	0.185	APK1	K2592+	Maximum	1.750
				400				270		
			APS2	K2592+	Minimum	0.111	APK2	K2592+	Minimum	1.712
				200				200		
C-Ⅱ	807	-0.022	APS3	K2592+	Maximum	0.222	APK3	K2592+	Maximum	1.789
				750				770		
			APS4	K2592+	Minimum	0.165	APK4	K2592+	Minimum	1.724
				475				430		
C-Ⅲ	807	0.033	APS5	K2592+	Maximum	0.206	APK5	K2592+	Maximum	1.786
				790				815		
			APS6	K2593+	Minimum	0.121	APK6	K2593+	Minimum	1.737
				170				095		
C-Ⅳ	0	0.033	APS7	K2593+	Maximum	0.138	APK7	K2593+	Maximum	1.761
				240				235		
			APS8	K2593+	Minimum	0.109	APK8	K2593+	Minimum	1.716
				330				320		

### 3.3 Association between accident rate and segment categorization

This study utilizes the F-test to examine differences in accident rates among various clusters of road segments based on perspective alignment features, and then compares these findings with traditional classification methods, as detailed in Table 5.

**Table 5:** Correlation analysis between segment clustering results and accident rate.

Cluster parameters	Step length(m)	Number of cluster	Cluster Order	Total length of cluster (km)	Accident rate (Acc /million vehicles km)		F	P-value
					Mean value	Standard deviation		
AAPS, AAPK	100	2	K1	53.3	0.211	0.228	10.033	0.002***
			K2	96.6	0.254	0.305		
		3	K1	58.8	0.264	0.328	5.423	0.005***
			K2	16.3	0.228	0.255		
			K3	74.8	0.222	0.242		
		4	K1	75.3	0.235	0.261	3.993	0.008***
			K2	48.1	0.261	0.333		
			K3	0.8	0.325	0.372		
			K4	25.7	0.206	0.218		
AAPS, AAPK	200	2	K1	53.8	0.213	0.208	5.898	0.015**
			K2	96	0.254	0.274		
		3	K1	70.8	0.22	0.212	3.409	0.034**
			K2	59.8	0.266	0.3		
			K3	19.2	0.225	0.224		
		4	K1	39.4	0.273	0.291	2.234	0.083*
			K2	39.4	0.216	0.206		
			K3	9.4	0.222	0.231		
			K4	61.6	0.235	0.255		
AAPS, AAPK	300	2	K1	99	0.252	0.25	3.451	0.064*
			K2	50.7	0.214	0.194		
		3	K1	78.9	0.245	0.245	1.128	0.325
			K2	46.5	0.246	0.239		
			K3	24.3	0.208	0.175		
		4	K1	31.5	0.224	0.211	1.936	0.123
			K2	68.4	0.264	0.264		
			K3	41.1	0.207	0.192		
			K4	8.7	0.254	0.211		
AAPS, AAPK	400	2	K1	95.6	0.248	0.231	1.127	0.289
			K2	54	0.224	0.194		
		3	K1	56.8	0.246	0.227	0.158	0.854
			K2	72	0.235	0.223		
			K3	20.8	0.236	0.182		
		4	K1	36	0.215	0.195	0.817	0.485
			K2	62	0.242	0.227		
			K3	43.2	0.259	0.233		
			K4	8.4	0.221	0.167		
Horizontal curve radius, Vertical gradient	-	4	CI	108.124	0.259	0.355	0.535	0.659
			CII	17.253	0.256	0.286		
			CIII	4.016	0.311	0.576		
			CIV	20.157	0.271	0.326		

When the step length is 100 m, 200m, and 300m, there is a significant difference in accident rates between different clusters of road segments (P-value < 0.1). Using traditional classification methods, the significance level of accident rates differences among categories is P = 0.659 > 0.1, indicating no significant difference in accident rates in classified segments based on two-dimensional alignment indicators. Overall, the significance level of differences in accident rates decreases with an increase in the number of clustering. Taking the classification results at a 200m step length as an example, the difference in accident rates is statistically significant for segments classified into 2 clusters (P = 0.015 < 0.05), followed by those classified into 3 clusters (P = 0.034 < 0.05), and then 4 clusters (P = 0.083 < 0.1). With the increase in road segment step length, the differences in accident rates gradually diminish. The most significant differences in accident rates are observed in the classification results for 100m step lengths, with P-values less than 0.01 for all step lengths. When the step length reaches 400m, there are no significant differences in accident rates among the clusters, regardless of the number of clusters into which the road segments are divided.

This observed phenomenon could be explained by returning to primary aim of this study, which is to extract alignment features from the driver’s perspective. During the process of driving, drivers concentrate on a segment of road ahead in the direction of travel, known as the visual range. They typically adjust their driving behavior based on the road conditions observable within visual range. Previous research indicates that at a design speed of 100 km/h, drivers can perceive traffic signs clearly at a visual recognition distance of approximately 300 m [32]. This distance is closely aligned with the segment length where the differentiation between accident classifications disappears in our study. Additionally, Chen et al [33] and Wang et al [34] conducted a relevant study investigating the relationship between road segment geometric features and safety level using different segment lengths. They found that the two-dimensional geometric features of road segments obtained using a 300-meter segment length had the most significant impact on safety surrogate measures (Chen, 2018; Wang, 2020). These similar conclusions further validate the reliability of our road segment classification results based on alignment perspective features.

In this study, alignment perspective features-based segment classifications reveal significant differences in accident rates, indicating a direct correlation between APS&APK and accident rates. Conversely, no significant correlations are found between segment classifications using traditional two-dimensional parameters and accident rates in the selected freeway. This underscores the premise that alignment perspective features are fundamental factors affecting the traffic safety [35] and highlights the limitations of traditional two-dimensional geometric parameters in safety analysis. The classification indicators proposed in this study reflect the continuous micro-changes in observed alignment features by drivers along the driving direction, providing more detailed information on alignment changes. This is something that traditional two-dimensional geometric parameters such as horizontal curvature and vertical gradient cannot achieve.

## 4 Conclusion

This study addresses the limitations of previous studies, which have failed to unify features from different viewpoints at the same cross-section when extracting quantitative characteristics from perspective views [36]. It utilizes similarity invariants to describe the road surface features within naturally occurring perspective images of driving video, offering a novel quantitative expression of alignment characteristics from the driver’s perspective. Two quantitative index related to alignment perspective views are proposed: alignment perspective skewness (APS) and alignment perspective kurtosis (APK). APS measures the overall inclination or symmetry of the road alignment shape from the driver’s perspective; while APK measures the sharpness or flatness at the top of the road alignment shape from the driver’s perspective.

Furthermore, the road segment classification method based on alignment perspective features demonstrates superior performance in analyzing differences in accident rates across road segments compared to traditional methods based on two-dimensional indicators. The classification method in this study can indicate overall differences in accident rates due to different road segment categories within a unit length of a road segment. In other words, the classification method proposed in this study exhibits greater sensitivity to accident rates than traditional methods, thereby potentially yielding more scientific and reliable results when applied to safety analysis and road segment classification management.

Since the indicators used for classification in this study can reflect the micro-changes in road features from the driver’s perspective, there is significant promise in analyze the dynamic micro-level effects of road geometry on driving behavior and driver psychology in future research. For instance, based on the continuous observability of alignment perspective features along the driving direction, it can enhance existing operational speed models based on two-dimensional longitudinal parameters. This could potentially offer a deeper insight into the micro-level impact of geometry on operational speed.

Nevertheless, several limitations should be noted for this study. This study concentrates solely on extracting alignment perspective features and conducting safety analyses in daylight conditions, it’s essential to recognize the significance of nighttime alignment perspective feature analysis for traffic safety. However, it’s hard to standardize perspective features in nighttime driving images due to challenges like poor lighting, limited visibility, and the influence of factors such as vehicle headlights and types. Consequently, our forthcoming efforts will be directed towards researching nighttime perspective feature extraction. This includes comparing and analyzing how perspective features differ between nighttime and daytime, measuring differences in safety risks, identifying hazards from road design flaws, and proposing new ideas to improve road safety.

Second, only a specific mountain freeway has been examined to assess alignment perspective features concerning traffic safety. It would be advantageous to extend this method to include additional freeways or other types of roads to investigate the spatial transferability of the proposed road segmentation method and its correlation with traffic safety.

In addition, this study is an initial exploration into the essential characteristics of alignment from the driver’s perspective, as well as road segment classification methods. At this stage, no correlation regression model or conversion model has been developed to link alignment perspective features with traditional two-dimensional geometric design parameters. Therefore, the findings of this study have operational limitations when applied to guiding alignment optimization designs under the existing design system. Moving forward, we will focus on seeking breakthroughs in this area, aiming to integrate alignment perspective features from the driver’s perspective into alignment design indicators from the design perspective, laying a foundation for guiding the geometric design of actual road projects.

## Supporting information

S1 File Provides the information related to alignment perspective features extraction models, segment categorization models, and details regarding the accident rates for various road alignment categories. (DOCX)
