# Supplementary material for: Exploring road safety using alignment perspective features in real driving images: A case study on mountain freeways
Source: PLoS One. 2024 Jun 17;19(6):e0305241. doi: 10.1371/journal.pone.0305241 (PMC11182566; doi:10.1371/journal.pone.0305241)

Supplementary information

FIG S1 Experimental vehicle and driving video collection perspective


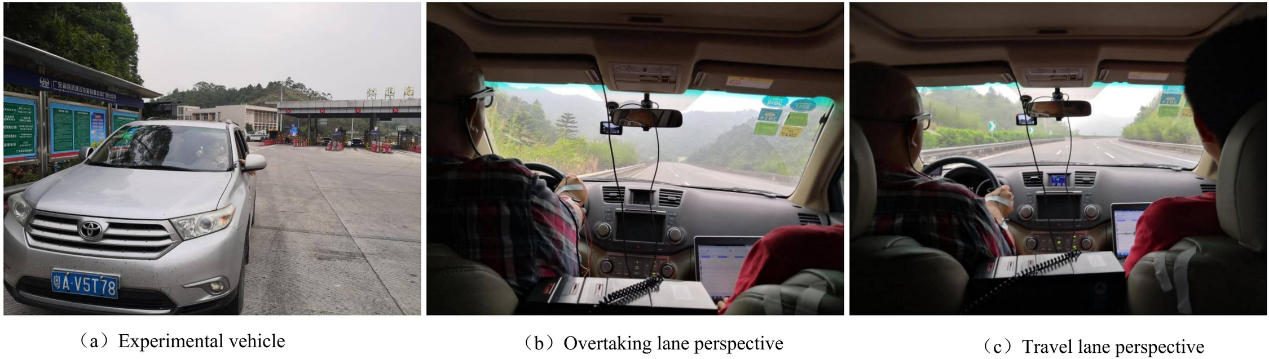


FIG S2 The Process of Extracting Perspective Features


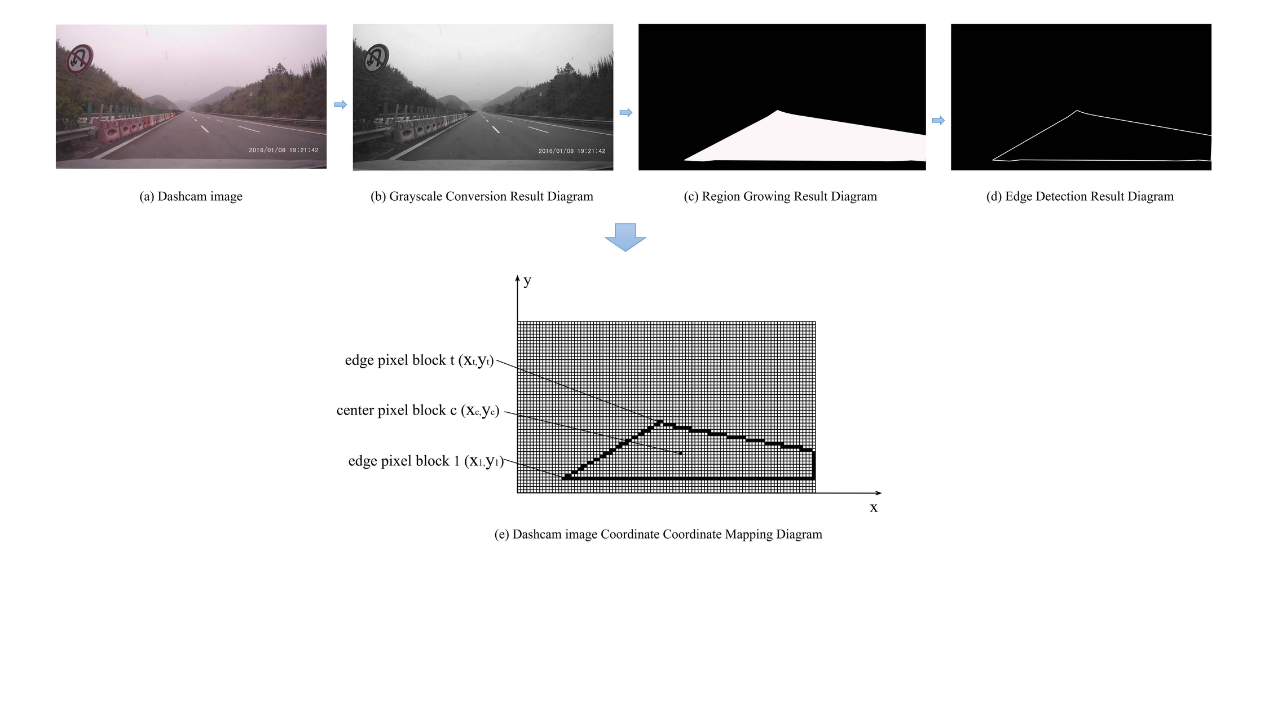


FIG S3 Statistical Chart of Perspective Feature Indicators


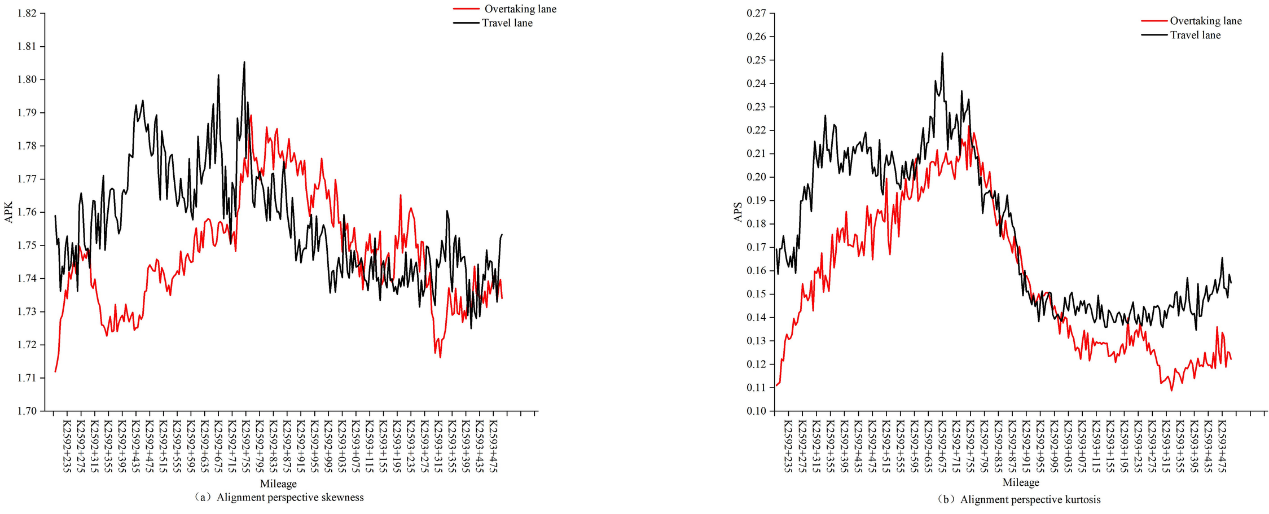


FIG S4 Scatter plot of clustering results for road segments at various step lengths


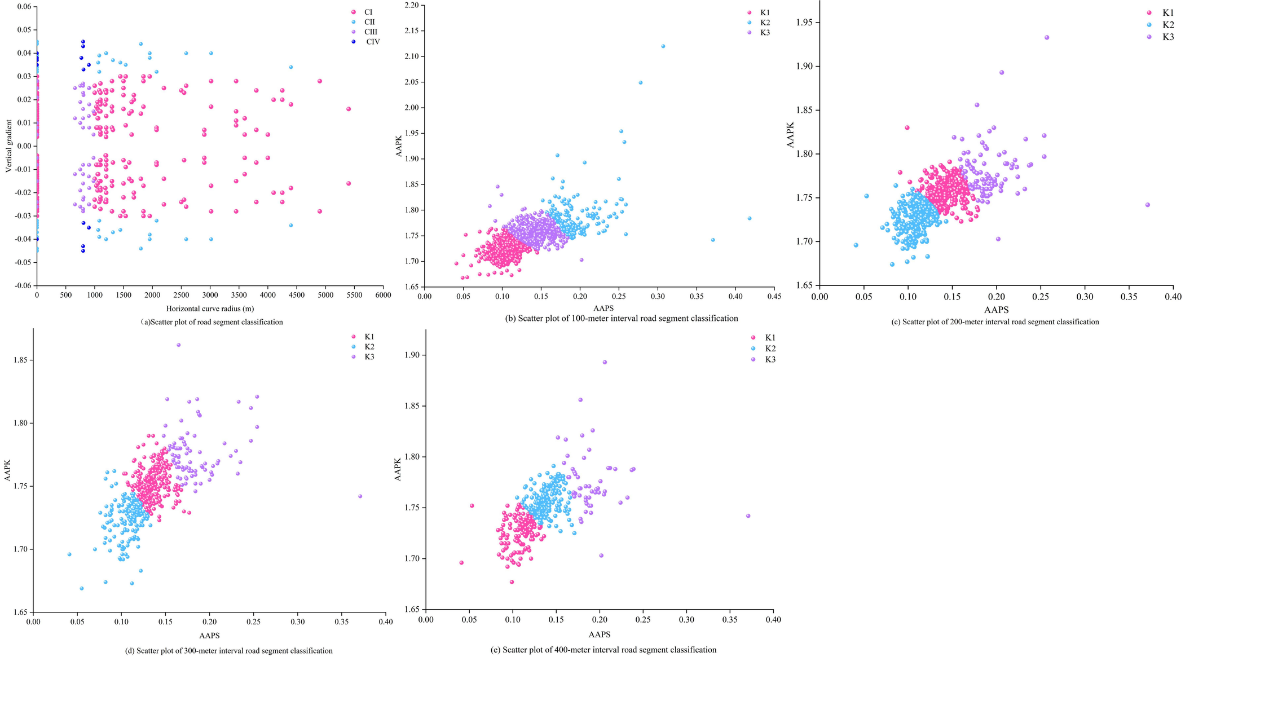


FIG S5 Cross segment diagrams at the extremities of perspective features


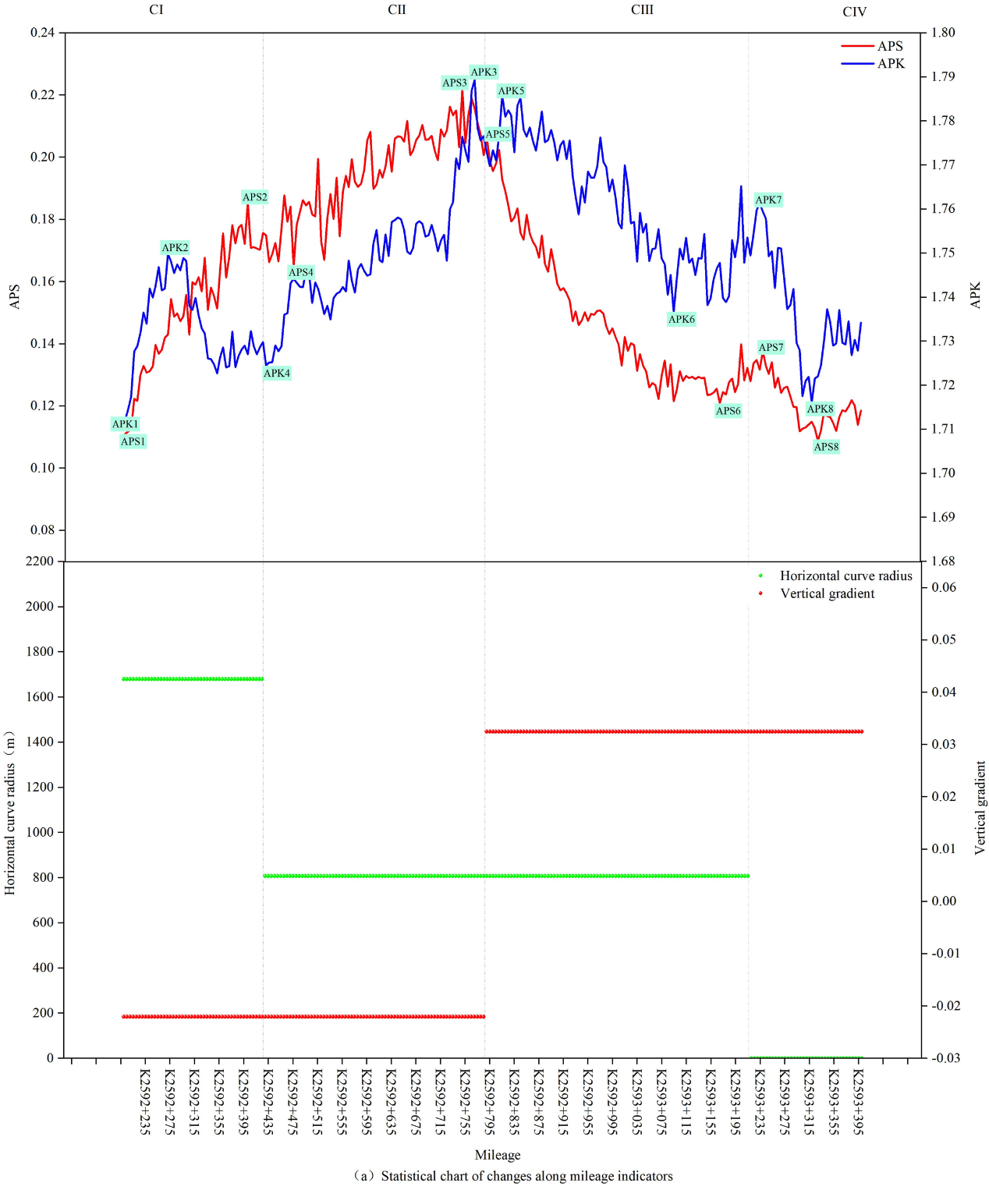


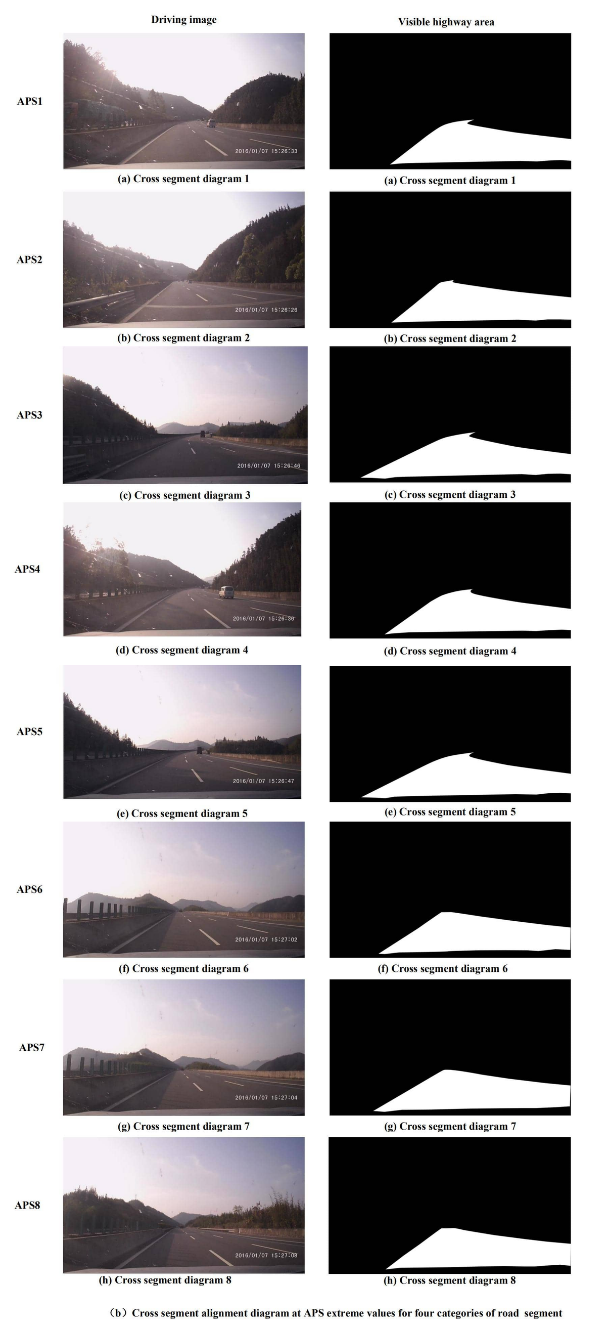

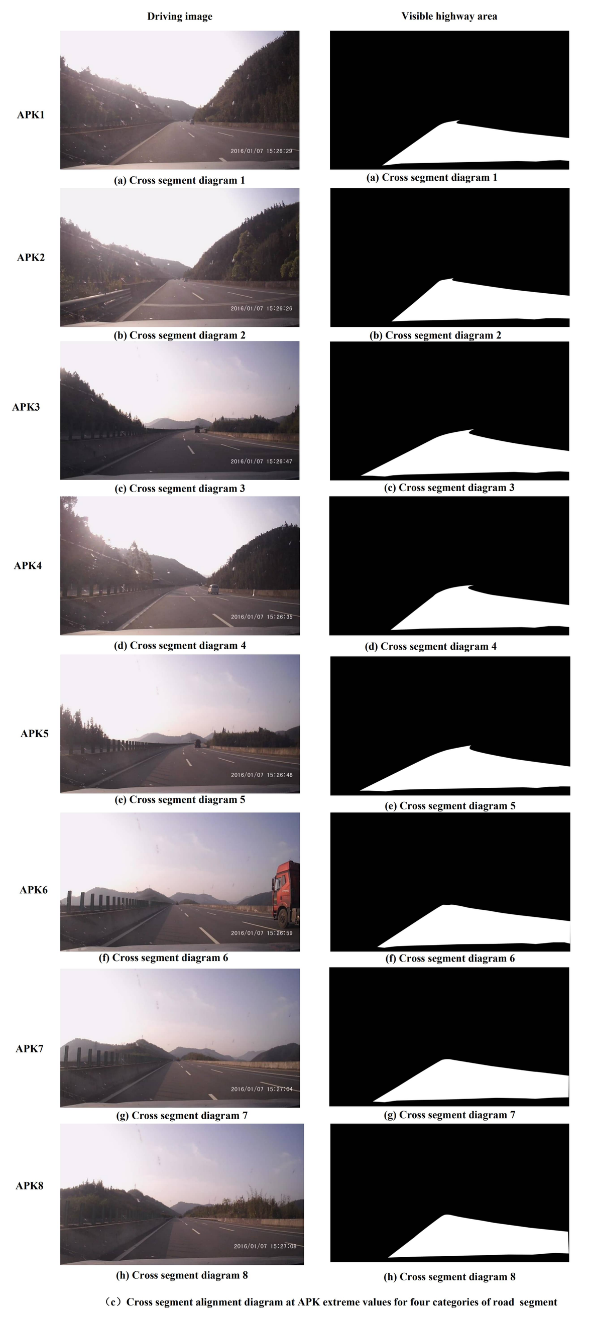

Supplement: S1 File — (DOCX) [file pone.0305241.s001.docx]
